# Supplementary material for: Trajectories of COVID-19: A longitudinal analysis of many nations and subnational regions
Source: PLoS One. 2023 Jun 23;18(6):e0281224. doi: 10.1371/journal.pone.0281224 (PMC10289358; doi:10.1371/journal.pone.0281224)
Supplement: S1 Appendix — (DOCX) [file pone.0281224.s001.docx]

# Appendix A

**Table A1.** Vaccination decay and Delta variant escape trajectories.

|  | **Country** |  | **Intervention efficacy** |  | **Decay**  **slope** |  | **Rebound slope** |  | **Post-vax steady state** |
| --- | --- | --- | --- | --- | --- | --- | --- | --- | --- |
|  |  |  | *η* |  | *δ* t_½_ |  | *r* t_2_ |  | log*I* SD |
|  |  |  | % |  | wk^-1^ wks |  | wk^-1^ wks |  | wk^-1^ wks |
| **Europe** | Austria |  | 60 |  | 0.48 1.44 |  | 0.60 1.16 |  | 2.5 ± 0.4 |
|  | Belgium |  | 45 |  | 0.50 1.39 |  | 0.51 1.36 |  | 2.9 ± 0.3 |
|  | Cyprus |  | 60 |  | 0.61 1.14 |  | 1.00 0.69 |  | 3.0 ± 0.4 |
|  | Czechia |  | 45 |  | 0.48 1.44 |  | 0.30 2.31 |  | 2.3 ± 0.6 |
|  | Estonia |  | 60 |  | 0.50 1.39 |  | 0.61 1.14 |  | 2.5 ± 0.5 |
|  | Finland |  | 50 |  | 0.32 2.17 |  | 0.48 1.44 |  | 2.4 ± 0.5 |
|  | France |  | 60 |  | 0.89 0.78 |  | 0.80 0.87 |  | 2.6 ± 0.5 |
|  | Germany |  | 60 |  | 0.54 1.28 |  | 0.47 1.47 |  | 3.0 ± 0.5 |
|  | Greece |  | 50 |  | 0.45 1.54 |  | 0.74 0.92 |  | 3.0 ± 0.5 |
|  | Hungary |  | 75 |  | 0.54 1.28 |  | 0.48 1.44 |  | 2.9 ± 0.5 |
|  | Israel |  | 60 |  | 0.67 1.03 |  | 0.59 1.17 |  | 2.5 ± 0.5 |
|  | Italy |  | 99 |  | 0.51 1.36 |  | 0.68 1.02 |  | 2.7 ± 0.4 |
|  | Netherlands |  | 50 |  | 0.52 1.33 |  | 0.6 1.2 |  | 2.4 ± 0.5 |
|  | Norway |  | 50 |  | 0.36 1.93 |  | 0.47 1.47 |  | 1.3 ± 0.5 |
|  | Slovenia |  | 90 |  | 0.76 0.91 |  | 0.44 1.58 |  | 2.7 ± 0.4 |
|  | Slovakia |  | 85 |  | 0.48 1.44 |  | 0.62 1.12 |  | 2.4 ± 0.7 |
|  | Spain |  | 80 |  | 0.47 1.47 |  | 0.70 0.99 |  | 2.5 ± 0.5 |
|  | Switzerland |  | 50 |  | 0.69 1.00 |  | 0.65 1.07 |  | 3.2 ± 0.5 |
|  | **UK** |  | 75 |  | 0.35 1.98 |  | 0.47 1.47 |  | 2.4 ± 0.5 |
|  | England |  | 66 |  | 0.34 2.04 |  | 0.48 1.44 |  | 3.1 ± 0.5 |
|  | Wales |  | 80 |  | 0.34 2.04 |  | 0.58 1.20 |  | 2.1 ± 0.7 |
|  | Scotland |  | 61 |  | 0.31 2.24 |  | 0.68 1.02 |  | 2.1 ± 0.4 |
|  | N. Ireland |  | 72 |  | 0.29 2.39 |  | 0.48 1.44 |  | 2.3 ± 0.4 |
|  | **mean**  **SD** |  | **64**  **15** |  | **0.50 1.52**  **0.15 0.45** |  | **0.58 1.27**  **0.15 0.33** |  | **2.6**  **0.5** |
| **Asia** | Japan |  | 99 |  | 0.5 1.3 |  | 0.6 1.3 |  | 2.7 ± 0.4 |
|  | Malaysia |  | 50 |  | 0.6 1.2 |  | 0.5 1.5 |  | 2.1 ± 0.5 |
|  | Singapore |  | 60 |  | 0.4 1.9 |  | 0.7 1.0 |  | 3.7 ± 0.4 |
|  | **mean**  **SD** |  | **70**  **26** |  | **0.50 1.47**  **0.10 0.38** |  | **0.60 1.27**  **0.10 0.25** |  | **2.8**  **0.4** |
| **US** | Connecticut |  | 75 |  | 0.55 1.26 |  | 0.61 1.14 |  | 2.7 ± 0.2 |
|  | Illinois |  | 83 |  | 0.50 1.39 |  | 0.58 1.20 |  | 2.8 ± 0.3 |
|  | Massachusetts |  | 99 |  | 0.49 1.41 |  | 0.77 0.90 |  | 3.5 ± 0.3 |
|  | N. Hampshire |  | 91 |  | 0.51 1.36 |  | 0.69 1.00 |  | 2.2 ± 0.2 |
|  | New Jersey |  | 44 |  | 0.80 0.87 |  | 0.55 1.26 |  | 3.2 ± 0.2 |
|  | New York |  | 84 |  | 0.47 1.47 |  | 0.52 1.33 |  | 2.8 ± 0.2 |
|  | Pennsylvania |  | 65 |  | 0.41 1.69 |  | 0.52 1.33 |  | 2.8 ± 0.4 |
|  | Rhode Island |  | 98 |  | 0.61 1.14 |  | 0.69 1.00 |  | 2.4 ± 0.3 |
|  | **mean**  **SD** |  | **80**  **18** |  | **0.54 1.32**  **0.12 0.24** |  | **0.62 1.15**  **0.09 0.16** |  | **2.8**  **0.3** |
|  | **mean** |  | **69** |  | **0.51 1.47** |  | **0.59 1.24** |  | **2.64** |
|  | **CI_95%_** |  | **62-75** |  | **0.46-0.56 1.3-1.6** |  | **0.55-0.64 1.1-1.3** |  | **2.48-2.80** |
