# Supplementary material for: Trajectories of COVID-19: A longitudinal analysis of many nations and subnational regions
Source: PLoS One. 2023 Jun 23;18(6):e0281224. doi: 10.1371/journal.pone.0281224 (PMC10289358; doi:10.1371/journal.pone.0281224)
Supplement: Attachment — Submitted filename: Review report for PONE-D-23-01297.pdf [file pone.0281224.s003.pdf]

Review report for PONE-D-23-01297

Trajectories of COVID-19: a longitudinal analysis of many nations and subnational Regions

David Burg, Jesse H. Ausubel.

The manuscript considers an interesting topic about COVID-19 for several countries in the world. However, this paper is not easy to understand due to errors in writing. The quality of the figures is not high standard and hard to get the information described. The following are some of the errors or unclear descriptions needed to improve.

1. Remove the dot at the end of title.
2. Line 120 on page 5: “Deaths can be thought of as a subset …and neglected for the purpose of this study.” If the deaths are significant, I do not think we can neglect. Can you provide evidence from the data you have about this?
3. Line 136 on page 5: $r^2 + (\delta + c)r + \delta c(1 - R_0)$ should be an equation following the text.
4. Line 158 on page 6: No Figure 1 is shown here but it is shown at the end. What are the initial values used in this figure?
5. Line 152: what is t_0? From Figure 1, it is the time exponential decay starting. Please give an explanation.
6. Line 153 on page 5: insert a space between δ and “can”.
7. Line 180 on page 7: what is “(Berkeley Madonna v8)”?
8. Line 196, page 8: the lines used in Fig. 2 for different countries are not readable and time intervals are not the same. The first two are between March 2020 and March 2021 but the last one is between March 2020 and February 2022. When we compare the varying rates, we may use the same time intervals.
