# Supplementary material for: Trajectories of COVID-19: A longitudinal analysis of many nations and subnational regions
Source: PLoS One. 2023 Jun 23;18(6):e0281224. doi: 10.1371/journal.pone.0281224 (PMC10289358; doi:10.1371/journal.pone.0281224)
Supplement: Attachment — Submitted filename: Review report for PONE-D-23-01297 R1.pdf [file pone.0281224.s005.pdf]

Review report for PONE-D-23-01297

Trajectories of COVID-19: a longitudinal analysis of many nations and subnational Regions

David Burg, Jesse H. Ausubel.

The manuscript considers an interesting topic about COVID-19 for several countries in the world. The revised version is improved in explanations and graphs. Except for the following issue, I did not find anything else needed to be fixed.

1. Line 158 on page 6: "The intrinsic growth rate constant, r, is solved for by the dominant root of the equation: $r^2 + (\delta + c)r + \delta c(1 - R_0)$" is incorrect. This was one of the comments for the initial submission, but this problem has not been fixed.
