# Supplementary material for: Trajectories of COVID-19: A longitudinal analysis of many nations and subnational regions
Source: PLoS One. 2023 Jun 23;18(6):e0281224. doi: 10.1371/journal.pone.0281224 (PMC10289358; doi:10.1371/journal.pone.0281224)
Supplement: Attachment — Submitted filename: Comments to the author.docx [file pone.0281224.s002.docx]

The authors examined transmission of COVID-19 using data from various countries. They observed the reduction, rebound, and convergence of confirmed cases according to government policies and suggested a mathematical model that could simulate these policies. It has been shown that the estimated values from this model agree with the observed phenomena. In particular, it was a notable result that, contrary to my expectations, the decay rates d and rebound rates r0 were similar in all countries and regions studied during the pandemic. The author has did a good work. hope my comments would help the authors to improve their work.

Major:

1. First, in the introduction section, it would be good to clarify the differences and novelties of this study from related studies. The model used in this study is a basic model used in virus dynamics. This model represents the intracellular spread of the virus in humans. It would be nice to have a detailed reason why this model was applied to the COVID-19 spread simulation. (I recommend introducing related studies, summarizing the results, and writing more prominently the advantages of using this model.)
2. It would be easier to see if there was a table that summarized the description of the parameters, the values, and references used in the model.
3. Regarding comment 2, it would be useful to explain what role a group of free virus particles('V' compartment) play in the COVID-19 spread model in this compartment model. In the general SIR model, it is assumed that an individual belonging to group S is infected by contact with an individual belonging to group I. In this study, however, it is not. The strength of this study is the use of the virus dynamics model for the spread of COVID-19, so it is necessary to explain how the compartment used in the model applies to the spread of COVID-19 in order to emphasize this. (It is a basic model, but it would be nice to describe the dynamics of this model in the Appendix or provide related references.)
4. Line 119 “uninfected people are being made available at a constant rate (\sigma)”.

Why is the number of uninfected people continuously increasing in the sentence above? (In general, sir-based models assume population growth at birth.)

5. How did you get the lockdown efficacy from Table 2 on page 12? This appears to be related to \eta in eq(1). However, this \eta represents not only lockdown, but overall intervention efficacy such as social distancing and vaccination. If the intervention efficacy is interpreted by limiting it to Lockdown, the estimated values in Table 2 are the optimal control solutions for \eta?

Minor:

1. It would be good to add a diagram representing Eq(1) (lines 124-126).
2. Github address on line 191 is not connected.
3. In Table A1 of Appendix, the post-vax steady state is shown as blank.
4. Since the figures in appendix are in black and white, it is difficult to distinguish between countries.

Positive:

1. I like table2, which summarizes the results for many countries.
2. Figure 6 shows the waves of COVID-19 in Israel well, and at the same time it is good to grasp the "characteristic time" at a glance.
