# Supplementary material for: Trajectories of COVID-19: A longitudinal analysis of many nations and subnational regions
Source: PLoS One. 2023 Jun 23;18(6):e0281224. doi: 10.1371/journal.pone.0281224 (PMC10289358; doi:10.1371/journal.pone.0281224)
Supplement: Attachment — Submitted filename: PLOSone responses2.docx [file pone.0281224.s006.docx]

We thank the reviewer for his comment. Below is our response.

**Reviewer #2:**

Minor:
1. Line 158 on page 6: “The intrinsic growth rate constant, r, is solved for by the dominant root of the equation: r^2+(δ+c)r+δc(1-R_0 )” is incorrect. This was one of the comments for the initial submission, but this problem has not been fixed.

Response: We thank the reviewer and apologies for missing this on the first round. It is now corrected:

r^2+(δ+c)r+δc(1-R_0)=0
